# Association between circulating neuregulin4 levels and diabetes mellitus: A meta-analysis of observational studies

**DOI:** 10.1371/journal.pone.0225705

**Published:** 2019-12-09

**Authors:** Yao Wang, Shuai Huang, Pei Yu

**Affiliations:** NHC Key Laboratory of Hormones and Development (Tianjn Medical University), Tianjin Key Laboratory of Metabolic Diseases, Tianjn Medical University Chu Hsien-I Memorial Hospital & Tianjin Institute of Endocrinology, Tianjin, China; Institute of Clinical Biochemistry, Clinical Molecular Medicine and Laboratory Medicine, ITALY

## Abstract

**Introduction:**

Neuregulin 4 (Nrg4) was proven as a brown fat-enriched secreted factor that can regulate glucose and lipid metabolism. However, the association between circulating Nrg4 levels and diabetes mellitus (DM) in human remains unclear. We conducted a meta-analysis to investigate association of circulating Nrg4 with DM.

**Methods:**

Observational studies comparing circulating Nrg4 levels in diabetes patients and health controls were included. Circulating Nrg4, correlation coefficients of clinical indices and circulating Nrg4 were pooled by meta-analysis.

**Results:**

Seven studies were included. The pooled results indicated there were no significant difference in the circulating Nrg4 between diabetes patients and controls (SMD = 0.18, 95%CI = -0.06 to 0.42, *P* = 0.143). However, diabetes patients had higher circulating Nrg4 than their controls in cross-sectional studies (SMD = 0.55, 95%CI = 0.36 to 0.73, *P*<0.001). None of the renal function and metabolic syndrome markers were correlated with circulating Nrg4, whereas the HbA1c and BMI were positively correlated (r_s_ = 0.09, 95%CI = 0.03 to 0.16, *P* = 0.005; r_s_ = 0.20, 95%CI = 0.07 to 0.34, *P* = 0.003; respectively).

**Conclusion:**

Our findings suggested circulating Nrg4 may play a role in in the development of DM in cross-sectional studies and circulating Nrg4 might be associated with imbalance in glucose metabolism and obesity.

## Introduction

It is well known that adipose tissue contributes to systemic energy balance and metabolic homeostasis. White adipose tissue (WAT) stores fat and interacts with the central nervous system and other peripheral tissues, brown adipose tissue (BAT) serves a role on defense against cold and energy excess [1–3]. Recently, increasing publications have indicated adipose tissue derived factors exert effects on the process of regulating system metabolism and BAT has been evidenced to have an endocrine function in regulating energy metabolism and balance [4–8]. According to rat tissue and brown adipogenesis transcriptome data, a list of brown fat-enriched secreted factors was confirmed [9]. Among these, neuregulin4 (Nrg4), which not only shows universal-expressed characteristic in multiple organs, but also concentrates in the brown adipose tissue (BAT), is central to regulate energy homeostasis and improve diet-induced metabolic disorders, including hepatic steatosis and insulin resistance [10]. Wang [9] reported that Nrg4 might activate the ErbB3/ErbB4 signaling in mice hepatocytes to coordinate glucose and lipid homeostasis in obesity. Ma [11] demonstrated that up-regulation of Nrg4 could improve insulin resistance (IR) and prevent weight gain via Nrg4 gene transfer in mice. Wang [12] showed that Nrg4 overexpression could improve the efficacy of mesenchymal stem cells (ADSCs) in ameliorating IR and other obesity-related metabolic disorders. Rosell and his colleagues suggested Nrg4 could be a crucial factor for promoting the browning process of white fat by sympathetic innervation of white adipose tissue (WAT) depot, thus activating the thermogenic functions, enhancing energy consumption, and reducing adiposity [13]. Taken together, these researches established the therapeutic potential of Nrg4 for treatment of obesity and associated disorders such as diabetes mellitus (DM). But the association between circulating Nrg4 levels and DM is not yet fully understood in humans, moreover, the results of previous studies on this relationship were not inconsistent. For these reasons, the aim of our meta-analysis was to investigate whether circulating Nrg4 levels were associated with DM and clinical indices of diabetes, renal function, metabolic syndrome and obesity in diabetes patients.

## Materials and methods

### Search strategy

We searched the PubMed, Embase, Web of Science databases from establishment to July 2, 2019 using the terms ‘‘diabetes” or ‘‘diabetes mellitus” or ‘‘DM” and ‘‘neuregulin 4” or ‘‘Nrg4” or ‘‘adipokine”. We selected only English-language, and the reference lists of identified publications were hand-examined, and the grey literature or ongoing relevant studies were searched on http://www.opengrey.eu/ and http://www.ntis.gov/.

### Study selection

Cross-sectional studies and case control studies comparing circulating Nrg4 levels in diabetes patients and health controls were included. The primary outcome was the association between circulating Nrg4 levels and DM. The second outcome was correlation coefficients between circulating Nrg4 levels and the measured clinical indices in subjects with DM. The clinical indices include DM markers (fasting plasma glucose (FPG); HOMA-IR, hemoglobin A1c (HbA1c)), renal function markers (estimated glomerular filtration rate (eGFR), creatinine), metabolic syndrome markers (systolic blood pressure (SBP), diastolic blood pressure (DBP), low-density lipoprotein (LDL-C), high-density lipoprotein (HDL-C), triglyceride (TG), total cholesterol (TC)), obesity markers (body mass index (BMI), waist circumference (WC)). Studies were excluded for the following reasons: publication of abstracts, duplicate data, overlapping population, unavailable data, literature reviews, animal experiment, and case report.

### Data extraction

The information extracted from each original study by two investigators (Y.W. and S.H.), the name of first author, publication year, country, the number of subjects, mean age, circulating Nrg4 levels and correlation coefficients between Nrg4 and the measured clinical indices dealt by spreadsheets of Microsoft Excel. If there were opposite views between the two investigators, the discrepancy would be resolved through discussion. Moreover, one of the investigators would email the authors of the enrolled articles to get more information if data were absent.

### Statistical analysis and quality assessment

Drafted on the basis of a preset protocol registered with PROSPERO 2018 (CRD42018103571), the present meta-analysis was conducted in line with the preferred reporting items for systematic reviews and meta-analyses (PRISMA) statement [14]. Stata 14.0 (Stata Corp, College Station, TX, USA) was used to carry out the meta-analysis. Odds ratios (ORs) and 95% confidence intervals (CIs) were calculated on pooled effects for dichotomous variables, while the standard mean difference (SMD) with 95% confidence intervals (CIs) were calculated for continuous variables. The *I*^2^ test was used to examine heterogeneity among the studies, *I*^2^ values of 25–50%, 50–75% and 75% are considered indicative of low, moderate and high heterogeneity, respectively [15]. If *I*^2^<50%, a fixed-effect model was conducted; otherwise, radom-effect model was used. Sensitivity and subgroup analyses were attempted to identify potential sources of the between-study heterogeneity if necessary. Subgroup analyses were conducted by region (Asia and non-Asia), age (matched or higher in DM), and study type (cross-sectional and case-control). Begg’s and Egger’s test contributed to publication bias. *P* <0.05 was regarded as statistically significant.

Besides, as for the association between circulating Nrg4 levels and clinical indices in diabetes patients, Spearman correlation coefficients were selected for present meta-analysis, because they are unaffected by monotonic transformations. Pearson correlation coefficients could transform into Spearman correlation coefficients by equation: r^s^ = 6/πsin^-1^ r^p^/2, where r^p^ represents the Pearson correlation coefficient and r^s^ represents the Spearman correlation coefficient [16]. Aiming to adjust the sampling distribution of the Spearman correlation coefficients, the Fisher transformation shown in equation: z = 1/2_*_[ln (1+r^s^)-ln (1-r^s^)] was performed to transform correlation coefficient to an almost normally-distributed variable fisher’s z with SE (SE = 1/√(n-3)), where n is the sample size of diabetes patients. After these conversion, forest plots could be established to show the summary fisher’s z (Z_s_) and its 95% confidence intervals (CIs) [17]. Subsequently, the Z_s_ was converted to the summary correlation coefficient (r_s_) by the transformation shown in equation: r_s_ = e^2Zs^-1/e^2Zs^_+_1.

Newcastle-Ottawa Quality Assessment Scale (NOS) was used to assess the quality of case-control studies [18], while, Agency for Healthcare Research and Quality (AHRQ) was appropriate for assessment of cross-sectional studies [19]. Disagreements were resolved by argumentum.

## Results

**Fig 1:**
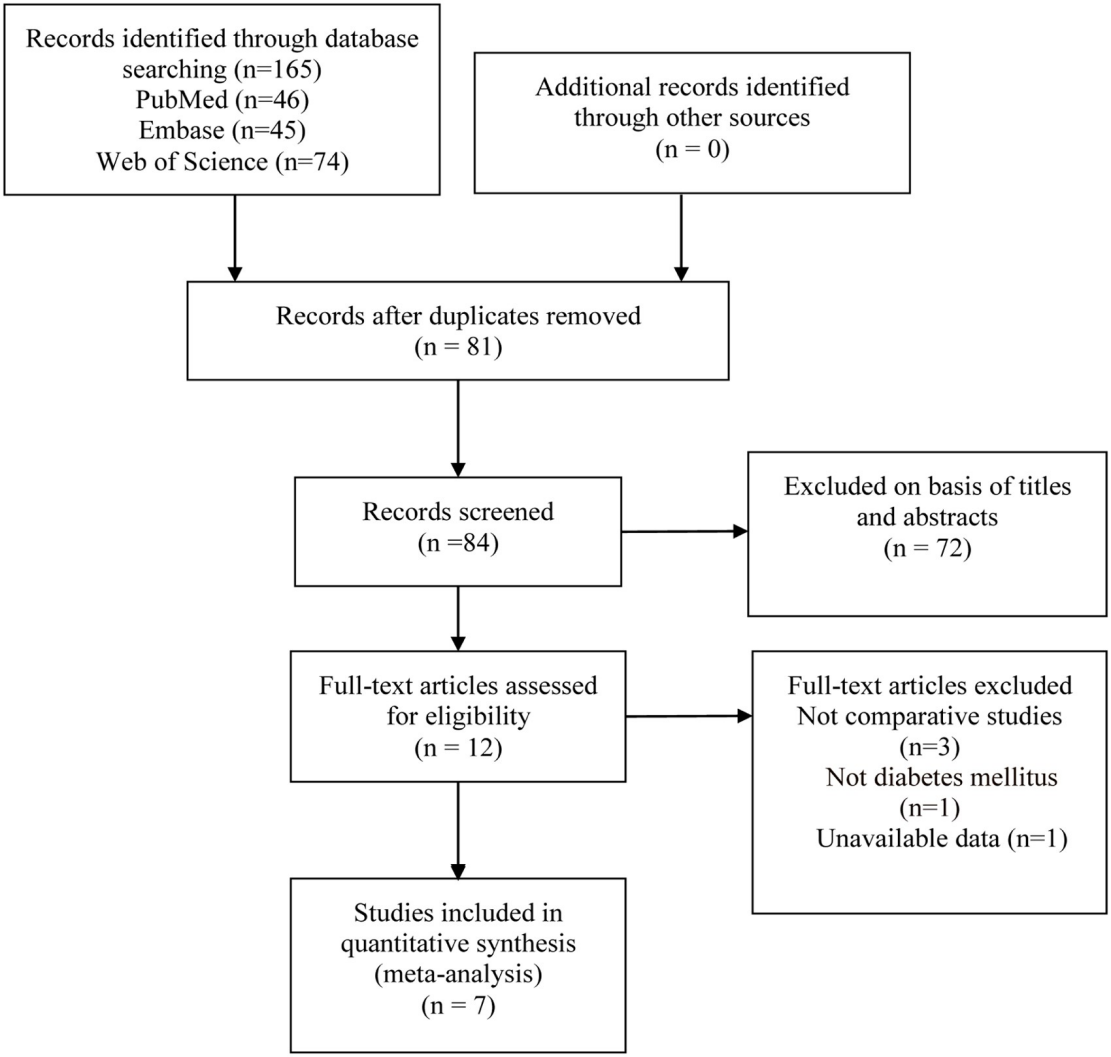
Identification of eligible articles.

Overall, 165 studies were identified in our initial search, of which 12 underwent full-text review (Fig 1). Finally, 7 studies [20–26] involving 2257 patients were identified and enrolled in the meta-analysis (Fig 1). Detailed information for another 5 excluded articles is accessible in S1 Table. The baseline demographics of these enrolled studies were shown in Table 1. Results of quality assessment of all included studies were relatively satisfying and shown in S2 Table.

**Table 1:** Characteristics of the studies included in the meta-analysis.

Study	Year	Country	Sample size^a^	Age(y)^a^	Nrg4 (ng/mL)^a^	Diabetes markers^r^			Renal function markers^r^		Metabolic syndrome markers^r^						Obesity markers^r^	
						FPG	HOMA-IR	HbA1c	eGFR	Creatinine	SBP	DBP	LDL-C	HDL-C	TG	TC	BMI	WC
Cai C [20]	2016	China	781/431	54.3/51.6	3.24/3.55	-0.094	-0.045	NR	NR	NR	-0.036	-0.045	-0.014	0.047	-0.051	-0.018	-0.095	-0.107
Chen LL [21].	2017	China	96/83	59.0/56.6	0.40/0.08	0.176	NR	0.096	-0.214	NR	0.127	0.134	0.09	-0.133	0.167	0.025	0.159	0.182
Jiang J [22]	2016	China	122/121	54.7/53.7	4.5/2.1	NR	NR	NR	NR	NR	NR	NR	NR	NR	NR	NR	NR	NR
Kang YE [23]	2016	Korea	57/59	50.5/51.1	0.778/0.579	0.23	0.192	0.183	-0.002	NR	NR	NR	-0.059	-0.162	0.191	-0.017	0.12	NR
Kurek Eken M [24]	2017	Turkey	63/64	29.78/29.03	3.57/1.85	0.775	0.348	-0.02	NR	-0.071	-0.11	-0.017	0.206	-0.24	0.387	0.138	0.35	NR
Kralisch S [25]	2017	Germany	74/74	31.0/28.9	3.0/3.5	-0.1	-0.041	-0.192	NR	-0.019	0.17	0.184	-0.001	0.107	-0.137	0.003	NR	NR
Zhang L [26]	2017	China	103/129	52.01/50.56	1.37/2.32	-0.2	-0.25	NR	NR	NR	NR	NR	NR	0.211	NR	NR	NR	NR

Abbreviations: BMI, body mass index; DBP, diastolic blood pressure; eGFR, estimated glomerular filtration rate; FPG, fasting plasma glucose; HbA1c, hemoglobin A1c; HDL-C, high-density lipoprotein cholesterol; LDL-C, low-density lipoprotein cholesterol; SBP, systolic blood pressure; Nrg4, neuregulin4; NR, not reported; TC, total cholesterol; TG, triglyceride; WC, waist circumference.

^a^ Values are all given as diabetes mellitus group/health control group;

^r^ Values are all given as Spearman correlation coefficients of circulating Nrg4 levels with clinical indices in diabetes patients in enrolled studies.

### DM and circulating Nrg4 levels

**Fig 2:**
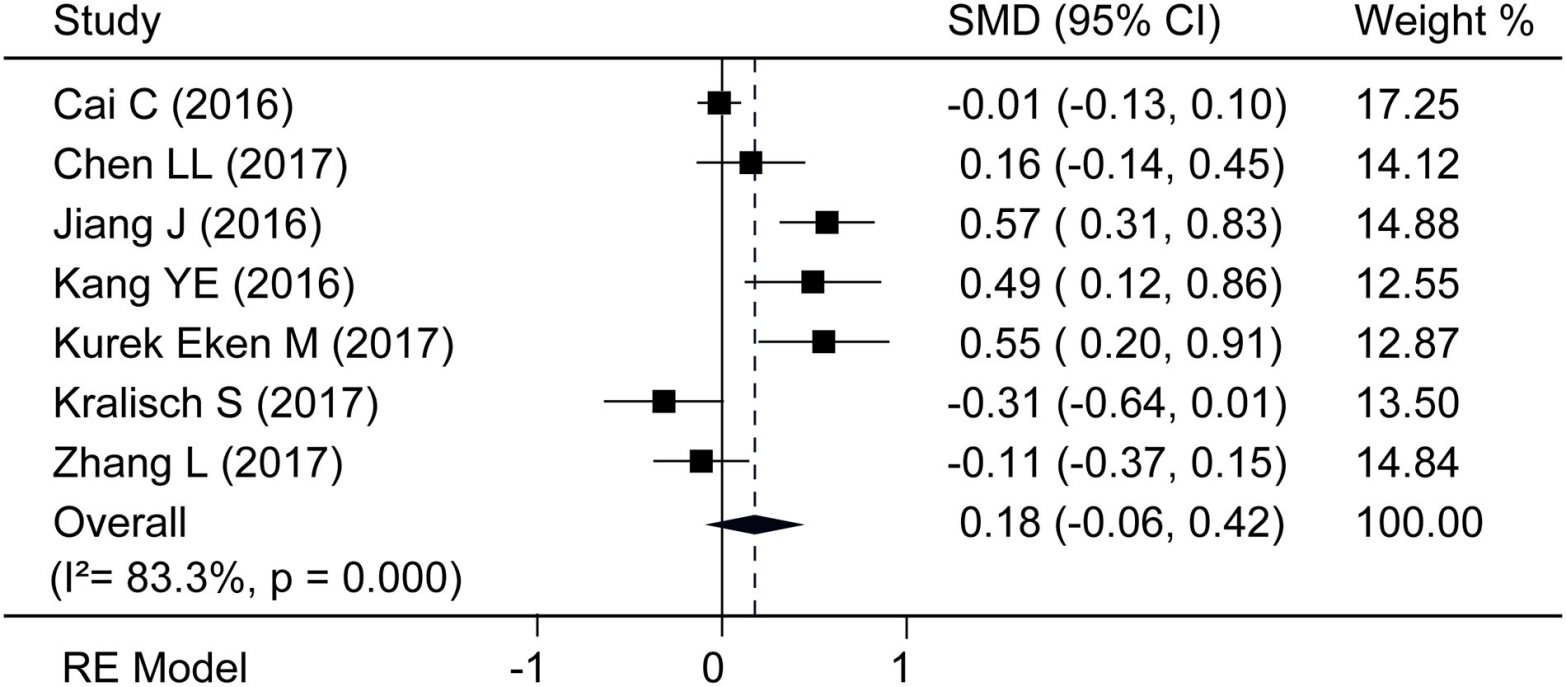
Forest plot of the association between circulation Nrg4 levels and DM; RE Model, random-effect model.

There were seven studies [20–26] regarding the association between circulating Nrg4 levels and DM, the *P* value of heterogeneity between these studies was significant, so we used the random-effect model indicating that there was no significant statistical difference between diabetes patients and their normal controls (SMD = 0.18, 95% CI = -0.06 to 0.42, *P* = 0.143) (Fig 2). The results of subgroup analyses by region (Asian[20–23,26] or non-Asian[24–25]), age (matched[21,24–26] or higher [20,22–23] in DM) study type (case-control [20–24,26] or cross-sectional[25]) are summarized in Table 2. A subgroup of cross-sectional study demonstrated that diabetes patients had higher circulating Nrg4 levels than their normal controls (SMD = 0.55, 95% CI = 0.36 to 0.73, *P*<0.001). Moreover, subgroup analysis according to region and age demonstrated that no significant difference of circulating Nrg4 levels between two groups was recorded. The sensitivity analysis presented a robust result that was not influenced by individual studies. Neither the Begg’s (*P* = 0.548) test nor the Egger’s (*P* = 0.316) test showed publication bias.

**Table 2:** Subgroup analysis of the association between circulating Nrg4 levels and DM.

	Studies	DM	Control	SMD	95% CI	*I*^2^
Countries						
Asia[20–23,26]	5	1159	823	0.20	-0.06 to 0.46	83%
Non-Asia[24–25]	2	137	138	0.18	-0.06 to 0.42	92.0%
Study type						
Cross-sectional [20–24,26]	6	1222	887	0.25	**0 to 0.50**	**83.1%**
Case-control [25]	1	74	74	0.18	-0.06 to 0.41	-
Age						
Matched [21,24–26]	4	336	350	0.33	-0.11 to 0.77	90.3%
Higher in DM [20,22–23]	3	960	611	0.06	-0.27 to 0.40	79.3%

DM, diabetes mellitus; Nrg4: neuregulin4; SMD: standardized mean difference. Bold values indicate SMD is significant between DM and the controls (p<0.05).

### Diabetes markers and circulating Nrg4 levels

**Fig 3:**
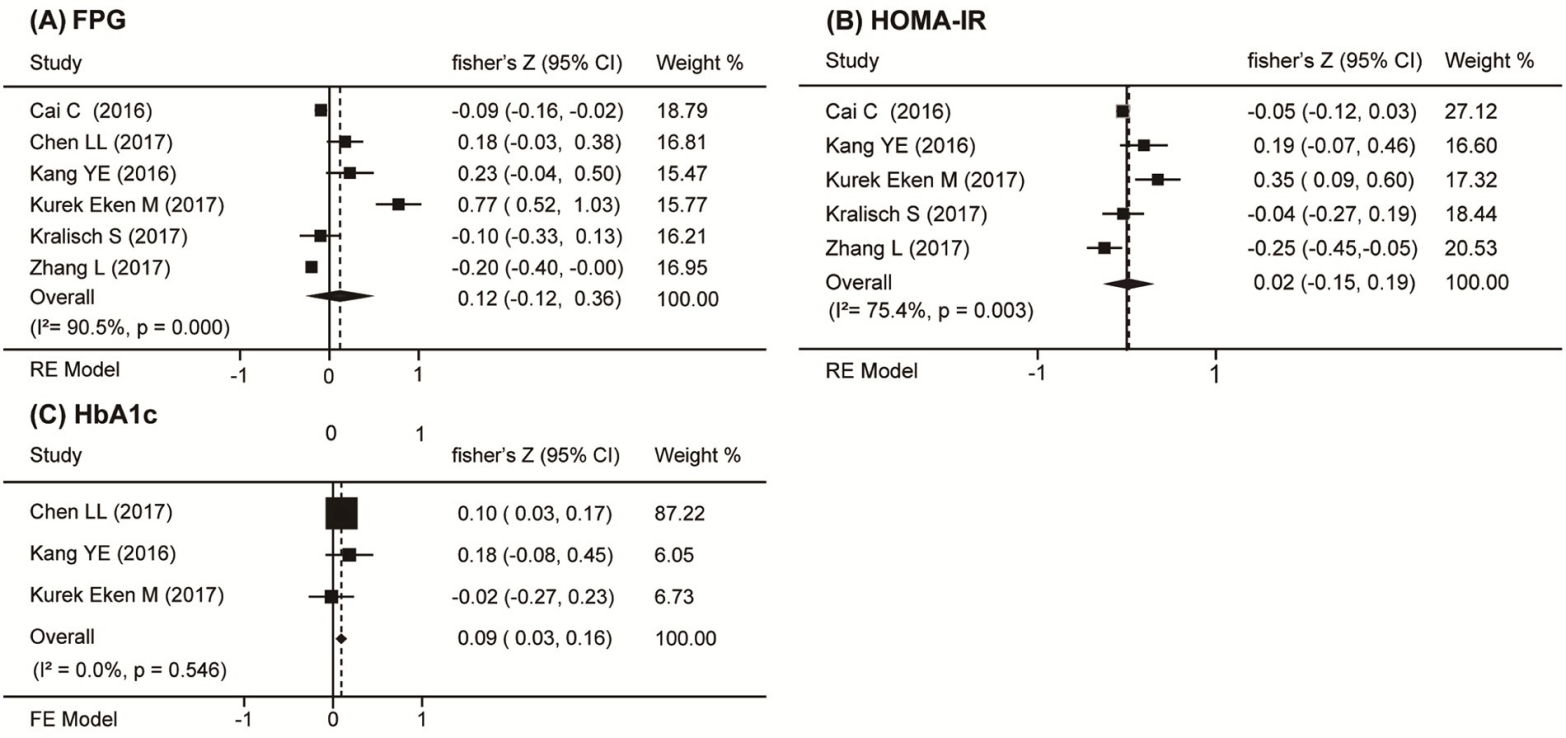
Forest plot of the association between circulation Nrg4 levels and diabetes markers in DM. Summaries are shown of the association circulation Nrg4 levels and (A) FPG, (B) HOMA-IR and (C) HbA1c; FE Model, fixed-effect model.

Six studies [20–21,23–26] presented data on the correlation coefficients of circulating Nrg4 levels and fasting plasma glucose (FPG) in diabetes patients, and five studies [20,23–26] reported HOMA-IR. Neither index was significantly correlated with circulating Nrg4 levels (r_s_ = 0.12, 95% CI = -0.12 to 0.36, *P* = 0.330 and r_s_ = 0.02, 95% CI = -0.15 to 0.19, *P* = 0.806, respectively) (Fig 3A and 3B). No publication bias was noted in these parameters. The sensitivity analyses of studies with significant heterogeneity didn’t show results sensitive to any individual studies, and the subgroup analyses did not discover the sources of the heterogeneity. Hemoglobin A1c (HbA1c) were measured in four studies [21,23–25]. The overall correlation coefficient was not statistically significant (r_s_ = 0.03, 95% CI = -0.11 to 0.17, *P* = 0.685). Subgroup analysis had not found the sources of the significant heterogeneity. Based on sensitivity analysis, the most influential study by Kurek Eken M [24] was excluded, this resulted in a summary coefficient coefficient of 0.09 (95% CI = 0.03 to 0.16, *P* = 0.005) (Fig 3C), neither the Begg’s (*P* = 1.000) test nor the Egger’s (*P* = 0.708) test showed publication bias.

### Renal function markers and Nrg4

**Fig 4:**
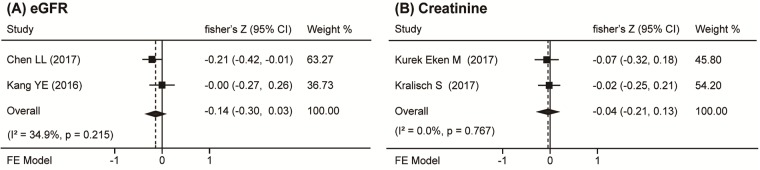
Forest plot of the association between circulation Nrg4 levels and renal function markers in DM. Summaries are shown of the association circulation Nrg4 levels and (A) eGFR and (B) creatinine.

The renal function markers estimated glomerular filtration rate (eGFR) and creatinine were each discussed in two of the included studies. It was found that eGFR and creatinine were no significantly correlated with circulating Nrg4 (r_s_ = -0.14, 95% CI = -0.30 to 0.03, *P* = 0.099 and r_s_ = -0.04, 95% CI = -0.21 to 0.13, *P* = 0.624; respectively) (Fig 4A and 4B). No publication bias was noted in these parameters.

### Metabolic syndrome markers and circulating Nrg4 levels

**Fig 5:**
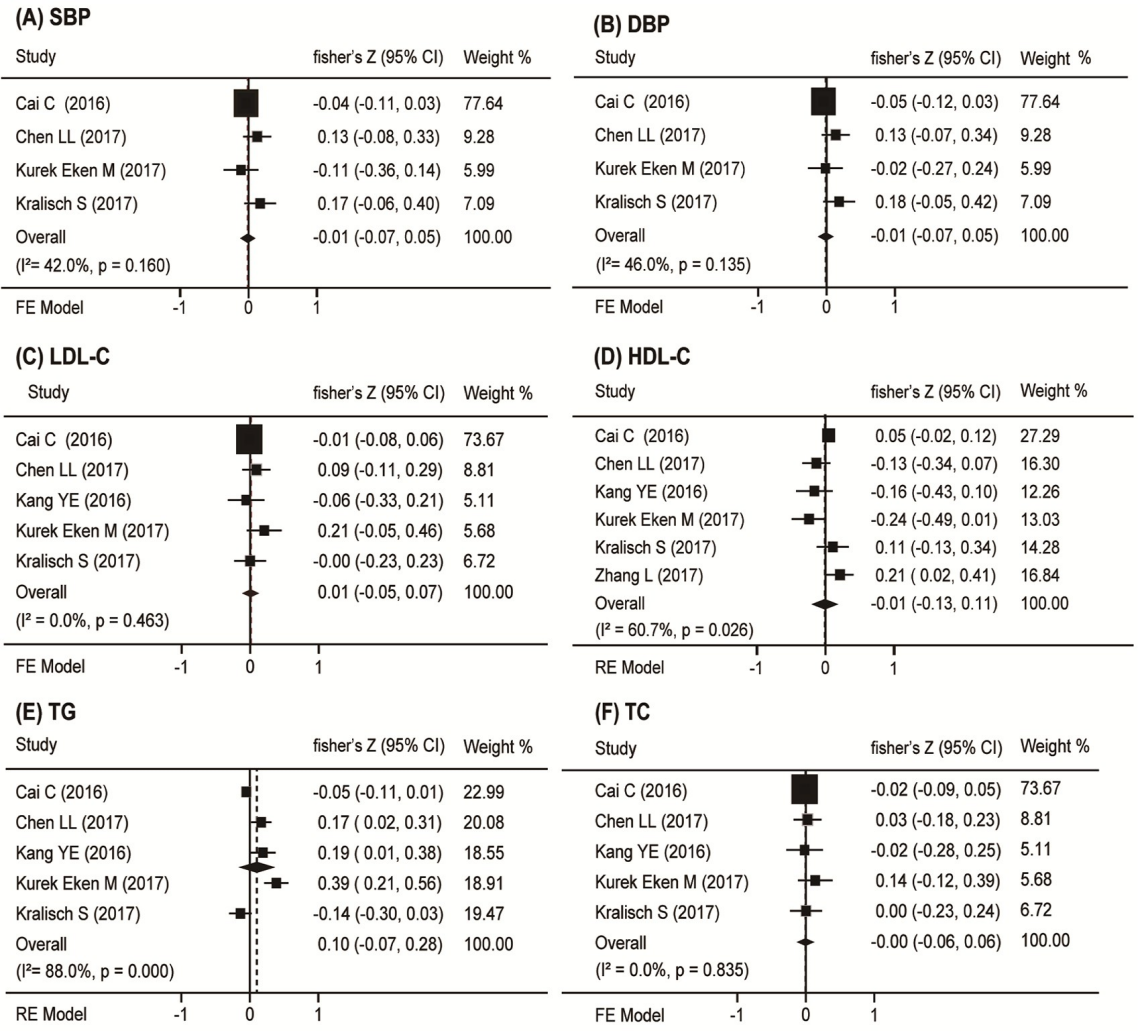
Forest plot of the association between circulation Nrg4 levels and metabolic syndrome markers in DM. Summaries are shown of the association circulation Nrg4 levels and (A) SBP, (B) DBP, (C) LDL-C, (D) HDL-C, (E) TG, (F) TC.

Blood pressure was reported in four studies [20–21,24–25]. Our meta-analysis showed that systolic blood pressure and diastolic blood pressure were not correlated with circulating Nrg4 levels (r_s_ = -0.01, 95% CI = -0.07 to 0.05, *P* = 0.735 and r_s_ = 0.05, 95% CI = -0.07 to 0.05, *P* = 0.740, respectively) (Fig 5A and 5B). Also, the low-density lipoprotein cholesterol (LDL-C) (r_s_ = 0.01, 95% CI = -0.05 to 0.07, *P* = 0.463) (Fig 5C), high-density lipoprotein cholesterol (HDL-C) (r_s_ = -0.01, 95% CI = -0.13 to 0.11, *P* = 0.882) (Fig 5D), triglycerides (TG) (r_s_ = 0.10, 95% CI = -0.07 to 0.28, *P* = 0.238) (Fig 5E) and total cholesterol (TC) (r_s_ = -0.00, 95% CI = -0.06 to 0.06, *P* = 0.900) (Fig 5F) were not significantly correlated with circulating Nrg4 levels. The sensitivity analyses of studies with significant heterogeneity did not show results sensitive to any individual studies, and the subgroup analyses did not discover the sources of the heterogeneity. No publication bias was noted in these parameters.

### Obesity markers and circulating Nrg4 levels

**Fig 6:**
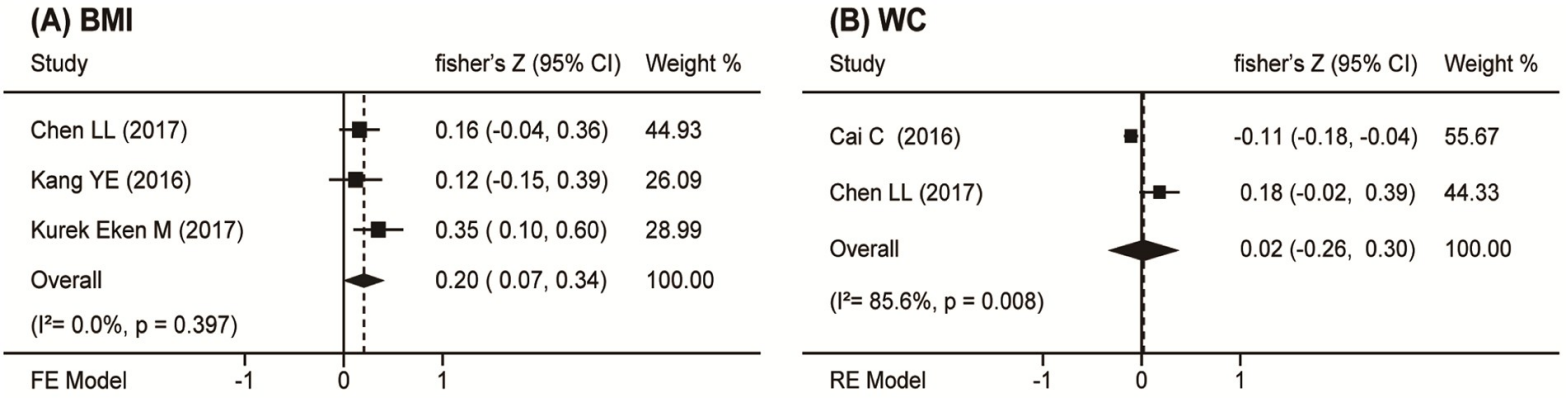
Forest plot of the association between circulation Nrg4 levels and obesity markers in DM. Summaries are shown of the association circulation Nrg4 levels and (A) BMI and (B) WC.

There were four included studies [20–21, 23–24] discussing about body mass index (BMI). The overall correlation coefficient was not significantly correlated with circulating Nrg4 levels (r_s_ = 0.11, 95% CI = -0.10 to 0.33, *P* = 0.301). Subgroup analysis did not find the sources of the significant heterogeneity. Based on sensitivity analysis, the most influential study by Cai C [17] was omitted, this resulted in a summary coefficient of 0.20 (95% CI = 0.07 to 0.34, *P* = 0.003) (Fig 6A). There was no publication bias found with Begg’s (*P* = 1.000) and Egger’s (*P* = 0.830) tests. Waist circumference (WC) was measured in two studies [20–21], and the overall correlation coefficient was also not correlated with circulating Nrg4 (r_s_ = 0.02, 95% CI = -0.26 to 0.30, *P* = 0.883) (Fig 6B). There was no publication bias found with Begg’s (*P* = 1.000) test.

## Discussion

It is well known that BAT could enhance energy expenditure through adjusting whole-body energy balance [27]. Meanwhile, activation of BAT is related to reduce adiposity, plasma lipid and glucose [28]. Besides, several recent studies indicated that BAT could secret endocrine factors working on other tissues to regulate systemic metabolism. One intriguing finding is Nrg4. Evidence showed that Nrg4 deficiency in mice and human obesity adipose tissue could aggravate insulin resistance and diet-induced lipid metabolic disorder [9]. But the correlation between circulating Nrg4 levels and DM in human has not been well characterized.

Our study is the first meta-analysis to investigate the association of Nrg4 with DM and clinical indices, indicating that circulating Nrg4 levels were not significantly associated with DM, but subgroup analysis according to study type demonstrated that circulating Nrg4 levels were higher in diabetes patients than their normal controls in the cross-sectional study group. Furthermore, neither renal nor metabolic syndrome markers was related to Nrg4, and HbA1c and BMI were positively associated with circulating Nrg4.

In our meta-analysis, circulating Nrg4 levels are not associated with the risk of DM. Contrastingly, Chen [21] demonstrated circulating Nrg4 levels was elevated in diabetes patients, similar results were concluded by Kang [23]. In contrast, some researchers reached the opposite conclusion [25, 26]. Therefore, meta-analysis concerning this relationship was carried out which showed no difference. We speculated the source of discrepancy might be from age, region, and study type. Therefore, subgroup analyses of them were performed. Our result of the subgroup of cross-sectional study raised the possibility that circulating Nrg4 levels might be a role in the development of DM, which was similar to Chen [21]. However, this result conflicted with those of a previous study reporting that Nrg4 stimulates insulin secretion and controls de novo liver lipid synthesis under metabolic stress in mice [9], and this study also revealed increased Nrg4 expression could improve insulin resistance and glucose intolerance, thus suggesting the prospect of treating obesity-related comorbidities such as diabetes. We harbored the idea that this phenomenon might be attributed to several reasons. Firstly, our result might embody a compensatory response to decreased expression of Nrg4 in adipose tissue or a kind of impairment in the Nrg4/ErbB signaling pathway by receptor resistance. That is to say, there may be “Nrg4 resistance” just like “insulin resistance” [21]. Secondly, although mainly released by adipose tissue, Nrg4 also could be secreted by other tissues such as liver, pancreas, stomach and muscle [29, 30]. Because the Nrg4 expression patterns might be evidently different among those tissues, the expression of Nrg4 in circulation under the condition of metabolic disorders could be distinct from that in adipose tissues. Thirdly, cold condition can affect the Nrg4 expression [31], leading to the different results under different temperatures. Unfortunately, such an analysis on this topic could not be performed owing to limited data. Finally, given that Nrg4 measurement might influence the results from different investigators, so the standardized Nrg4 assays are necessary.

The indices of the renal function and metabolic syndrome were not correlated with circulating Nrg4 levels in diabetes patients. Whereas the meta-analysis suggested that circulating Nrg4 levels were positively correlated with one of the glucose parameters (HbA1c), this finding was similar to some researchers [21, 23]. They concluded that the theory of “Nrg4 resistance” contributes to this correlation. Besides, although Nrg4 preserves glucose homeostasis in mice hepatocytes by activation of ErBb3/ErBb4 signaling, the universal distribution characteristic of ErBb3/ErBb4 signaling may lead to a totally different biological role of Nrg4 in other sites of human body [9, 32, 33]. On the contrary, Wang and Zhang [9, 26] argued that circulating Nrg4 levels were negatively correlated with glucose parameters. They explained that Nrg4 could stimulate insulin release from islet cells and improve insulin resistance under metabolic stress in mice [9, 34]. Taken together, the exact mechanism of Nrg4 in regulating metabolism has not been illuminated and requires further study.

Additionally, a positive correlation between adiposity proxies and circulating Nrg4 levels was confirmed in the meta-analysis. Intriguingly, Wang’s study encapsulated a negative correlation between obesity and adipose Nrg4 mRNA [9], and Cai [20] found that circulating Nrg4 was negatively associated with indices of obesity. Whereas Dai [35] showed that circulating Nrg4 was not correlated with obesity indices, similar results were found by Zhang [26]. Furthermore, Chen and Kang’s works were confirmed by our study [21, 23]. Expression of Nrg4 in each unit of adipose tissue, together with the total fat mass, forms a delicate balance and therefore gives an explanation to the discrepancy. In other words, although expression of Nrg4 in one unit adipose tissue decreases in obesity [9], the total fat mass should rise in obese subjects, which would increase the overall expression of Nrg4 through the whole body [21]. Thus the eventual expressing direction of Nrg4 for the whole body in obesity would be determined by the interaction of these two opposite notions, and each of the three different results might be reasonable.

In our meta-analysis, although we observed higher levels of circulating Nrg4 in diabetes patients in the cross-sectional studies, only one case-control study investigated this correlation [25], which suggested no significant association between circulating Nrg4 levels and DM. Since insufficient data on other study types, it was still inconclusive whether there was significant relationship of circulating Nrg4 levels and DM. Therefore, large prospective studies are required to confirm whether circulating Nrg4 levels are associated with the risk of DM in the future. Furthermore, standardized Nrg4 binding assays and analysis of Nrg4 expression pattern in multiple organs are necessary.

Our meta-analysis had some strengths. First of all, we evaluated the association of circulating Nrg4 levels with the risk of DM and glucose-related/non-glucose-related parameters in diabetes patients in the round, which added to the evidences on the role of circulating Nrg4 in human metabolism. Second, our meta-analysis could avoid some potential bias, because that results of stratification according to age and region still demonstrated no significant association between circulating Nrg4 levels and DM. Several limitations cannot be avoided in our meta-analysis. Firstly, the limited studies on this correlation could influence the strength of our meta-analysis conclusions. Secondly, the most of studies were based on cross-sectional data with a limited sample size, and given its cross-sectional design, it could not confirm the causal association between circulating Nrg4 levels and DM. Thirdly, the enrolled articles were all observational studies. The possibilities of selection and confounding bias could not be avoided. Indeed, diabetes patients could differ from their health controls in terms of economic power, access to healthcare services, attention to their own health and quality of our life. Finally, the included studies were lack of more useful markers of the metabolic syndrome, such as Visceral Adiposity Index (VAI) and Lipid Accumulation Product Index (LAP), which are more realistic than HOMA, BMI or WC. Therefore, more powerful original research with new and realistic markers of metabolic syndrome are warranted to elucidate the exact role of circulating Nrg4 in the development of DM in larger samples and in other ethnic groups.

## Conclusions

In summary, although our meta-analysis suggested circulating Nrg4 levels didn’t play a role in the process of DM, subgroup of the cross-sectional study revealed that circulating Nrg4 is higher in diabetes patients. As for clinical indices in diabetes patients, the glucose and adiposity parameters were positively associated with circulating Nrg4. The exact role of the circulating Nrg4 levels in the development of DM still needs to be further identified.

## Supporting information

S1 Checklist PRISMA checklist. (DOC)

S1 Table Reasons why 5 studies were excluded after full-text review. (DOC)

S2 Table Results of quality assessment. (DOCX)
