# Supplementary material for: Association between circulating neuregulin4 levels and diabetes mellitus: A meta-analysis of observational studies
Source: PLoS One. 2019 Dec 9;14(12):e0225705. doi: 10.1371/journal.pone.0225705 (PMC6901220; doi:10.1371/journal.pone.0225705)
Supplement: S1 Table — (DOC) [file pone.0225705.s002.doc]

| Reasons | Excluded studies |
| --- | --- |
| Not comparative studies | Yan P(2018); Wang GX(2014); Chen Z (2017) |
| Not diabetes mellitus | Dai YN(2015) |
| Unavailable data | Yan PJ(2017) |

Reasons why 5 studies were excluded after full-text review.
