# Supplementary material for: Association between circulating neuregulin4 levels and diabetes mellitus: A meta-analysis of observational studies
Source: PLoS One. 2019 Dec 9;14(12):e0225705. doi: 10.1371/journal.pone.0225705 (PMC6901220; doi:10.1371/journal.pone.0225705)
Supplement: S2 Table — (DOCX) [file pone.0225705.s003.docx]

| Author | Year | Country | Selection | Comparability | Outcome | Score |
| --- | --- | --- | --- | --- | --- | --- |
| Kralisch S | 2017 | Germany | **** | ** | *** | 9 |

**Quality of the 1 case-control study.**

Article quality was assessed as follows: low quality = 1–3; moderate quality = 4–6; high quality = 6–9.

**Quality of the 6 cross-section studies.**

| **Item Author** | Cai C | Chen LL | Jiang J | Kang YE | Kurek Eken M | Zhang L |
| --- | --- | --- | --- | --- | --- | --- |
| 1) Define the source of information (survey, record review) | Yes | Yes | Yes | Yes | Yes | Yes |
| 2) List inclusion and exclusion criteria for exposed and unexposed subjects (cases and controls) or refer to previous publications | Yes | Yes | Yes | Yes | Yes | Yes |
| 3) Indicate time period used for identifying patients | Yes | Yes | Yes | No | No | Yes |
| 4) Indicate whether or not subjects were consecutive if not population-based | Yes | Yes | Yes | Yes | Yes | Yes |
| 5) Indicate if evaluators of subjective components of study were masked to other aspects of the status of the participants | Yes | Yes | Yes | Unclear | Yes | Yes |
| 6) Describe any assessments undertaken for quality assurance purposes (e.g., test/retest of primary outcome measurements) | Yes | Yes | Yes | Yes | Yes | Yes |
| 7) Explain any patient exclusions from analysis | No | No | No | No | No | No |
| 8) Describe how confounding was assessed and/or controlled. | Yes | Yes | Yes | No | Yes | Yes |
| 9) If applicable, explain how missing data were handled in the analysis | No | No | No | No | No | No |
| 10) Summarize patient response rates and completeness of data collection | Yes | Yes | Yes | Yes | Yes | Yes |
| 11) Clarify what follow-up, if any, was expected and the percentage of patients for which incomplete data or follow-up was obtained | No | No | No | No | No | No |
| Score | 8 | 8 | 8 | 5 | 7 | 8 |

Article quality was assessed as follows: low quality = 0–3; moderate quality = 4–7; high quality = 8–11.
